# Production of Large-Ring Cyclodextrins by Amylomaltases

**DOI:** 10.3390/molecules27041446

**Published:** 2022-02-21

**Authors:** Kuakarun Krusong, Abbas Ismail, Karan Wangpaiboon, Piamsook Pongsawasdi

**Affiliations:** 1 Structural and Computational Biology Research Unit, Department of Biochemistry, Faculty of Science, Chulalongkorn University, Phyathai Rd., Patumwan, Bangkok 10330, Thailand; abbas.i@chula.ac.th (A.I.); karan.wa@chula.ac.th (K.W.); 2 Starch and Cyclodextrin Research Unit, Department of Biochemistry, Faculty of Science, Chulalongkorn University, Phyathai Rd., Patumwan, Bangkok 10330, Thailand; piamsook.p@chula.ac.th

**Keywords:** amylomaltase, cyclization, 4-α-glucanotransferases, large-ring cyclodextrin, starch

## Abstract

Amylomaltase is a well-known glucan transferase that can produce large ring cyclodextrins (LR-CDs) or so-called cycloamyloses via cyclization reaction. Amylomaltases have been found in several microorganisms and their optimum temperatures are generally around 60–70 °C for thermostable amylomaltases and 30–45 °C for the enzymes from mesophilic bacteria and plants. The optimum pHs for mesophilic amylomaltases are around pH 6.0–7.0, while the thermostable amylomaltases are generally active at more acidic conditions. Size of LR-CDs depends on the source of amylomaltases and the reaction conditions including pH, temperature, incubation time, and substrate. For example, in the case of amylomaltase from *Corynebacterium glutamicum*, LR-CD productions at alkaline pH or at a long incubation time favored products with a low degree of polymerization. In this review, we explore the synthesis of LR-CDs by amylomaltases, structural information of amylomaltases, as well as current applications of LR-CDs and amylomaltases.

## 1. Introduction

4-α-Glucanotransferases (4αGTases; EC 2.4.1.25) catalyze a hydrolysis of an α-1,4-linkage and a transfer of a (1,4)-α-d-glucan to an acceptor. The intermolecular transglycosylation or disproportionation reaction results in longer chain linear oligosaccharides, while the intramolecular transglycosylation or cyclization reaction—a dominant feature in this group—produces large-ring cyclodextrins (LR-CDs) or cycloamyloses (CAs). Members of 4αGTases include amylomaltases from microorganisms and disproportionation enzymes (D-enzymes) from plants and algae, as well as the bacterial cyclodextrin glucanotransferases (CGTases, EC 2.4.1.19). 4αGTases belong to the glycoside hydrolase GH13, GH57, and GH77 families as classified by the Carbohydrate Active Enzymes (CAZy) database [1].

LR-CD or CA is a cyclic (1,4)-α-d-glucan polymer consisting of nine or more glucose units, a higher degree of polymerization (DP) than well-known cyclodextrins (α, β and γ-CD or CD6, CD7, and CD8). LR-CDs can be produced from starch or linear amylose by an enzymatic reaction of 4αGTases, especially the amylomaltases and D-enzymes. They were first described by Pully and French in 1961 [2]. Since then, several 4αGTases have been reported to produce LR-CDs with DP ranging from 16 to more than 100. Nonetheless, CGTase can also produce LR-CDs from starch as minor side products, while the major products are CD6-8 [3,4]. It was reported that engineered CGTase from *Bacillus sp*. G-825-6 could produce mainly CD8-CD12 [5]. Although CD6-CD8 are widely used in food, cosmetic, and pharmaceutical industries to stabilize or enhance solubility of the guest molecules [6,7,8,9], LR-CD applications are limited due to small quantities obtained during synthesis and lack of efficient separation techniques.

A few articles reviewing 4αGTases have been published recently. Nakapong et al., 2022 focused on heterologous expression of 4αGTases including CGTase and amylomaltase for overproduction and beneficial properties for industrial applications [10]. Leoni and co-workers emphasized thermostability of amylomaltases from the extremophiles [11]. Ahmad et al., 2015 provided a review of structural similarities and mechanism of thermostable 4αGTases in comparison with other starch processing enzymes from other glycoside hydrolase (GH) families [12]. Herein, production of LR-CDs by amylomaltases and the current applications of LR-CDs and amylomaltases are discussed.

## 2. Sources and Biochemical Properties of Amylomaltase

Amylomaltases (AMs) catalyze four reactions including: (i) hydrolysis of α-glucosidic bonds in α-1,4-d-glucan; (ii) disproportionation or a transfer of a (1,4)-α-d-glucan to an acceptor; (iii) cyclization or the synthesis of LR-CD; and (iv) coupling or the hydrolysis of LR-CD. Generally, AM possesses low hydrolysis and coupling activities. Similarly, D-enzyme does not show activity on purified CA, unless glucose was added as an acceptor, resulting in smaller cyclic and linear products [13]. Kim et al., 2021 compared activities of AMs from *Deinococcus geothermalus* (*Dg*AM), *Thermus scotoductus* (*Ts*AM), and *Escherichia coli* (*malQ* or *Ec*AM) [14]. They reported that *Ec*AM exhibited more than 100-fold lower cyclization activity in comparison with *Dg*AM and *Ts*AM. Meanwhile, *Dg*AM and *Ts*AM showed similar kinetic results for disproportionation and cyclization activity, and both were equally capable to produce LR-CDs from amylose, with CD7 and CD27 as major products. Moreover, *Ts*AM showed about 5-fold higher transglycosylation/hydrolysis ratio compared to *Dg*AM. *Ec*AM also demonstrated relatively low disproportionation activity compared with the others.

AMs have been found in several microorganisms. In *E. coli*, AM or *malQ* is required in maltose/maltodextrin metabolism [15], as well as glycogen formation and degradation [16,17]. Besides the extremely thermophilic amylomaltases, a halophilic amylomaltase from halophilic archaeon *Haloquadratum walsbyi* has been discovered and characterized. This AM could efficiently exhibit starch transglycosylation activity in broad range of NaCl concentrations. This discovery might be promising for modifying starch in high ionic strength reaction and a direction for improving ionic strength tolerance of other AMs [18].

In plants, D-enzyme may involve with starch metabolism and chain length modification [19]. Repression of disporportionating enzyme 1 (*St*DPE1) and disproportionating enzyme 2 (*St*DPE2) altered malto-oligosaccharide content, starch content, and photosynthetic activity in potato [20]. It was suggested that D-enzyme plays an important role in degradation of linear glucans in *Arabidopsis* leaves [21].

Despite the fact that a large number of AMs have been effectively produced in *E. coli* hosts, some food and pharmaceutical applications may demand safer manufacturing. Thus, some AMs were studied to express in GRAS (generally recognized as safe) hosts such as *Bacillus subtilis* and *Saccharomyces cerevisiae* to be served in the applications in those fields [10]. *Ta*AM, which had been successfully expressed in *B. subtilis*, had its safety assessed in mice, demonstrating that it was sufficiently safe for food applications [22]. The optimum temperatures of characterized AMs are commonly around 60–70 °C for thermostable AMs and 30–45 °C for AMs from the mesophiles and plants. Halophilic AM was reported to exhibit optimal temperature at 40 °C [18]. However, some AMs from extreme thermophilic bacteria and archaea demonstrated higher optimum temperature such as the archaea *Pyrobaculum aerophilum* and *Pyrobaculum calidifontis*, with high disproportionation activity at 95 °C and 80 °C, respectively [11,23,24]. The ability to function at very high temperature make the thermostable AMs distinctly attractive for use in the synthesis of modified starch in high temperature reactions, such as for producing thermoreversible gel. Apart from that, a hyperthermophilic bacterium *Aquifex aeolicus* produced thermostable AM that exhibited maximal activity at 90 °C and retained 70% of its original activity after 30 min at 90 °C [25]. More common producers of AM are among *Thermus* species where the optimum temperature is conserved around 60–75 °C [26,27,28,29,30]. It is worth noting that a particular mutation on AM gene resulted in an increase in optimal temperature and half-life, compared to the wild type as illustrated by C446S mutated AM from *Streptococcus agalactiae*, where the optimum temperature increased by 5 °C from 40 °C and showed 3-fold increase in half-life at 45 °C [31]. Similarly, the mutated A460V enzyme from *Corynebacterium glutamicum* (*Cg*AM) exhibited 5 °C increase in optimum temperature which made it more thermostable compared to the wild type. The mutation resulted in increased catalytic efficiency and gave 46% higher product yields at 40 °C [32]. However, mutation of the same *Cg*AM at Tyr172 to produce Y172A mutant did not change the optimum temperature, but altered the LR-CD product pattern when compared to that of the wild type enzyme [33].

The optimum pH for AM to exhibit the maximum activity is around pH 6.0 to pH 7.0. Thermostable AMs tend to function at more acidic conditions, such as pH 5.5, by AM from *Thermus aquaticus* [26] and *Thermus thermophilus* [29]. Although most of AMs from mesophiles prefer neutral to basic conditions around pH 7.0 and above, one unique glycoside hydrolase family 77 AM from *Borrelia burgdorferi* was found to exhibit activity at pH 5.5 [34]. On the other hand, mutagenesis on *Cg*AM resulted in N287Y mutant, which exhibited a +0.5 unit increase of pH from the optimum and a decrease in disproportionation activity, compared to that of the wild type. The mutation resulted in increased thermostability; moreover, it also interrupted the hydrophobic and ionic interactions [35]. The increase in pH optimum around +0.5 unit was also observed in A460V *Cg*AM which demonstrated in the increase of product yield at long incubation time [32]. Table 1 summarizes sources of AMs and their optimum conditions.

## 3. Overall Structure of Amylomaltase

**Figure 1:**
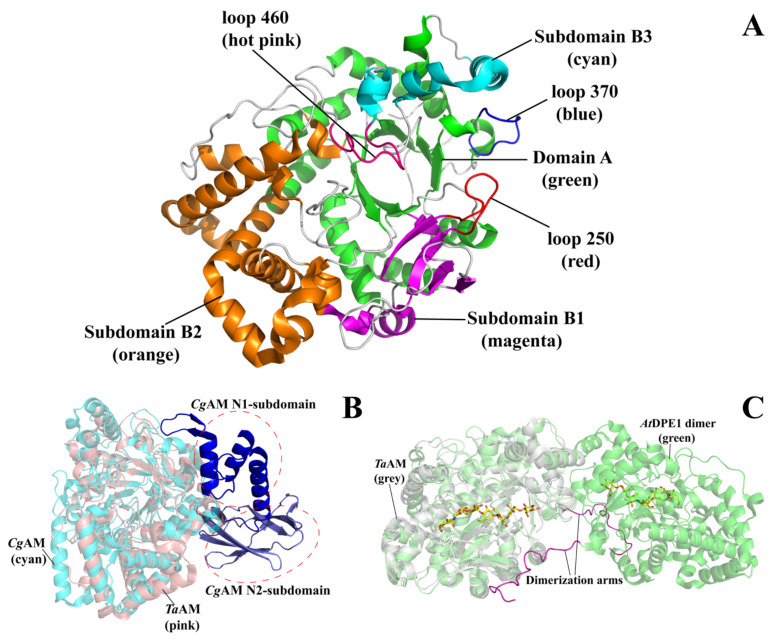
**The overall structure of AM.** Panel (**A**) presents the structure of *Ta*AM (PDB: 1CWY). Panel (**B**) is the structure of *Cg*AM (PDB: 5B68) superimposed with *Ta*AM and shows N1 and N2 subdomains. Panel (**C**) is the dimeric *At*DPE1 (PDB: 5CSU) superimposed with *Ta*AM.

The sequences of bacterial AMs were mostly classified into Glycoside hydrolase family 77 (GH77) and minor members in GH13 [1,11,44]. However, at least two enzymes from *Thermococcus litoralis* and *Archaeoglobus fulgidus* producing LR-CDs were discovered and categorized as a member of Glycoside hydrolase 57 (GH57) because of their distinguished structure and catalytic residues [45,46,47]. This review herein focuses on basic structure and mechanism of GH77 AM. The first deposited three-dimensional structure was the AM from *Thermus aquaticus* (*Ta*AM) [48], producing CAs larger than 22 mer [26]. The structure of *Ta*AM can be divided into two main domains, namely domain A and B. The subdomains B1, B2, and B3 are generated from insertion loops of (α,β)_8_ barrel structure of domain A (Figure 1A). In comparison with CGTase that mainly produces small ring cyclodextrins (SR-CDs) with 6–8 mer, AM showed fewer number of domains [49,50]. *Ec*AM [51] and *Cg*AM [52] share only 30% identity of protein sequences but both of them similarly represent an additional N-terminal domain, so-called domain N (Figure 1B). The subdomains N1 and N2 are linked by a short linker of nine amino acid residues. The β-sandwich of the subdomain N2 is similar to immunoglobulin fold, while the Greek key or jelly roll structure of the subdomain N1 is frequently found in carbohydrate-binding module as shown in various lectin structures. Domain A is responsible for the catalytic functions, while domain B involves in substrate binding. The subdomain N2 also interacts with subdomain B2 and plays a role in substrate binding. Mareček, F. et al. suggested that, based on bioinformatics, this N-domain might participate in α-glucan binding and probably lead to establish a new family of carbohydrate binding module (CBM) [44]. Nonetheless, further experiments are needed to confirm this notion.

It has been reported that the *Cg*AM and *Ec*AM could exist as oligomers (dimers or tetramers), depending on ionic strength [52]. Conversely, D-enzymes from *Arabidopsis thaliana* (*At*DPE1) [53] and the potato *Solanum tuberosum* [54], which show comparable actions to bacterial AMs, are present in a dimeric form. As shown in Figure 1C, the monomeric *At*DPE1 possesses a long N-terminal part as a dimerization arm for engagement (Figure 1C). Apart from DPE1, plants also produced DPE2 which is a cytosolic DPE isoform. This isoform specially contains two putative carbohydrate-binding modules (CBM20) at the N-terminus. In addition, the catalytic domain sequence is also divided into two parts as there is an insertion of 173 amino acid residues between the area of nucleophilic and acid/base-catalytic residues [55,56]. Unfortunately, no three-dimensional structure of DPE2 is available for structural analysis.

**Figure 2:**
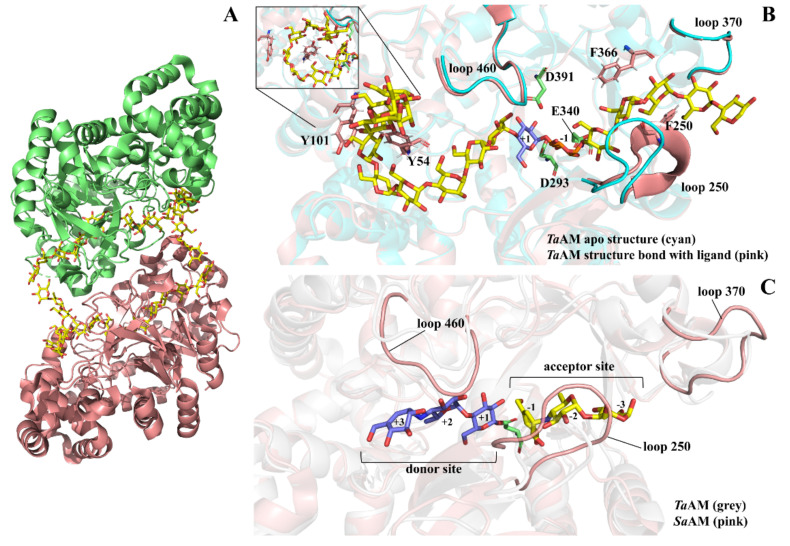
**Substrate binding tract of AM.** Panel (**A**) is an asymmetric structure of *Ta*AM co-crystallized with 34 mer cycloamylose (PDB: 5JIW). Panel (**B**) is a closed up active site of *Ta*AM bound with the glucan. The bound ligand structure (PDB: 5JIW) is superimposed with apo-structure (PDB: 1CWY). Panel (**C**) shows active site of *Sa*AM that presents acarbose-derived glycosyl-enzyme (blue) in the donor site and another ligand (yellow) in the acceptor site, while the D295, a nucleophile residue is in a light green color. *Sa*AM is superimposed with apo-structure of *Ta*AM (PDB: 1CWY).

Figure S1 in Supplementary Materials shows sequence alignment of AMs and D-enzymes. The catalytic triad residues of *Ta*AM consist of D293, E340, and D395 as a nucleophile, acid-base catalyst, and transition state-stabilizer, respectively, as evidenced by covalently bound intermediate of *Thermus thermophilus* AM (*Tt*AM) [57], *Streptococcus agalactiae* AM (*Sa*AM) [31], *At*DPE1 [53], and potato D-enzyme [54]. The whole substrate-binding tract of AMs was most completely illustrated by co-crystallization of *Ta*AM with 34 mer CA, presenting as an asymmetric dimer [58] (Figure 2A). The active site of AMs reveals a long track that is compatible with 16 glucose residues of amylose molecule (Figure 2B). The bound glucan chain interacted with *Ta*AM via 32 hydrogen bonds and 18 hydrophobic interactions. The crucial residues interacting with the ligand are demonstrated in Supplementary Materials Figure S2.

The active site of AMs contains two carbohydrate-binding sites; donor and acceptor sites in the opposite directions. This clearly confirms by the structures of *At*DPE1 [53] and *Sa*AM [31] that show intermediate donor molecule covalently linked with the nucleophile residues in donor site and also has another acceptor saccharide molecule occupied in another site of tract as shown in Figure 2C. The 460 loop insertion on subdomain B1 facilitates substrate binding in the middle of the active site. Y54 and Y101 between the (β/α)_8_-barrel subdomain A and subdomain B2 provide hydrophobic clamp as a turning point of the glucan chain for cyclization at + 7 position of the donor site, while F250 in the 250 loop on subdomain B3 at the acceptor site provides sugar tongs for facilitating transglycosylation and cyclization. Besides the 250 loop, the acceptor binding is also supported by the 370 loop on domain A. The loop forms hydrogen bond at + 4, + 5, and + 6 of the acceptor molecule via D369, D370, and E373, respectively (Figure 2B).

## 4. Cyclization Mechanism of Amylomaltase

**Figure 3:**
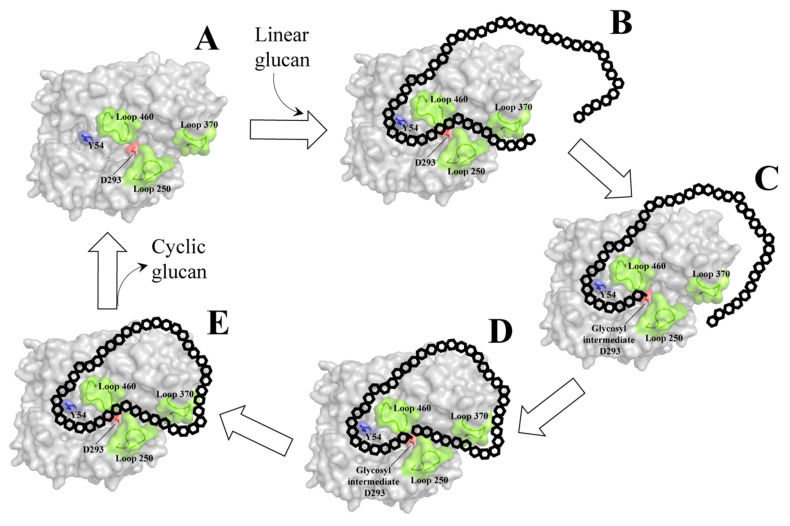
**Schematic illustration showing the cycle of mechanism of AMs**. (**A**) Apoenzyme. (**B**) Linear glucan is bound in the active site of AM. The bound glucan was cleaved between +1 and −1 subsite and then form glycosyl intermediate as shown in (**C**). (**D**) Non-reducing end of the bound glucan is folded back to the acceptor site. (**E**) Cyclic glucan is formed and then released before the AM reforms into its initial state. The schematic structure is illustrated based on *Ta*AM (PDB: 1CWY). The important loops (250, 370 and 460 loops) are in green. The acid/base catalytic residue D293 and the recognition site around Y54 are shown in red and blue, respectively.

Cyclization mechanism of AMs has been proposed based on *Ta*AM [58] and *At*DPE1 [52]. Initially, a glucan chain is engaged to the active site and it is cleaved between −1 and +1 position rings. The residual chain at the acceptor site is removed from the active site, while the remaining glucan chain is held in the glycosyl-intermediate form via the nucleophile residue. The non-reducing end of the held glucan is then folded back to the acceptor site, facilitated by the 250 and 370 loops. The glycosyl intermediate is transferred to the acceptor molecule via α-1,4-linkage. Finally, the product is released from the enzyme’s active site, while the enzyme is reformed to the initial state for the next cycle of catalysis (Figure 3).

## 5. Large-Ring Cyclodextrin Production by Amylomaltase

Cyclization reactions to produce LR-CDs or CAs were observed in AM, where the smallest LR-CDs reported to be produced is CD16 while the largest is more than CD100 [13,25]. As mentioned in the Introduction part, 4αGTase from GH13, GH57, and GH77 which include AM and CGTase are able to catalyze the cyclization of amylose to produce LR-CDs. Terada et al. [59] demonstrated the synthesis of CD9-CD60 by CGTase of GH13 using synthetic amylose as substrate, despite the LR-CDs were subsequently converted to smaller CDs at the later stage of reaction. The LR-CDs composed of 9 to more than 100 units of glucans were also produced by CGTase from *Bacillus* sp. and CGTase mutants successfully produced LR-CDs with minimum size of CD8 [5,60,61,62]. Meanwhile, 4αGTase from GH57 named GTase57 was also reported to produce LR-CDs with minimum DP of 17 using amylose as substrate [47].

Since 1996, many studies have reported the use of AM and members of GH77 to produce LR-CDs as illustrated in Table 2. Takaha and co-workers demonstrated the production of LR-CDs by potato D-enzyme on synthetic amylose substrate [13]. The products were illustrated to be resistant to hydrolysis by glucoamylase and had DP range from 17 to several hundreds. Meanwhile, a study from Terada and co-workers [26] was the first to discover the production of LR-CDs by bacterial AM. Similar to potato D-enzyme, use of AM from thermophilic bacterium *T. aquaticus* ATCC 33923 on synthetic amylose was found to produce LR-CDs, despite having minimum DP of 22. Over the years, several other thermophilic AMs have been reported to produce LR-CDs, but with different size. For example, *Tf*AM produced LR-CDs with the DP range of 22 to 60 [27], while *Ae*AM and *Dg*AM produced the DP range of 16 to 50 [25] and 5 to 37, respectively [14]. Meanwhile, studies on AMs from mesophilic bacteria [36,63,64] and plants [40] are also increasing. Recently, Tumhom and co-workers reported the biochemical characterization on AM from mesophilic *S. agalactiae*, where *Sa*AM was observed to catalyze the production of LR-CDs with the DP range of 22 to 50 when using pea starch as substrate [31].

Cyclization reaction to produce LR-CDs involved intramolecular transglycosylation, whereby the reaction conditions, incubation time, substrate, substrate pretreatment, and gene mutagenesis would impact the product yields. For instance, the thermostable AMs work best at high temperature range from 50 to 90 °C [14,25,27], meanwhile AMs from the mesophiles and D-enzymes prefer a low temperature within the range of 35 to 40 °C [31,36,40,41,65]. Reactions on higher temperature tend to produce higher DP products, compared to the reactions on low temperature as demonstrated by thermostable AM. The pH also determined the production of LR-CDs, where for examples, *Tf*AM prefers more acidic condition at pH 5.0 to produce highest yield while *Sa*AM and *Cg*AM prefer less acidic condition at pH 6.0 [27,31,63]. Shifting pH of reaction toward more alkaline resulted in higher amount of smaller LR-CDs as demonstrated in *Cg*AM study [66].

Several previous studies reported the influence of incubation time in cyclization reaction to the LR-CD yield and the principal size distribution. For example, *Tf*AM on pea starch produced smaller LR-CDs (CD22–CD29) in short incubation (2–4 h), while larger LR-CDs with DP 30-36 and 37-44 were observed in longer incubation (6–24 h) [27]. On the contrary, *Cg*AM demonstrated larger LR-CDs (CD31) at shorter incubation time at 30 min, while the LR-CDs became smaller at 4 and 24 h incubation time where the principal products were CD27-28 and CD25, respectively [36]. On the other hand, the amounts of LR-CDs were observed to be increased along with longer incubation time [67].

Substrate preference of a particular AM also affects the LR-CD production yield and LR-CD size. It was reported that *Cg*AM preferred raw tapioca starch than soluble tapioca starch, where *Cg*AM on raw tapioca starch produced a 2.8 times higher yield [63]. Both substrates gave same pattern of LR-CD size range (CD22-CD54), but different major products; CD27 and CD25-CD27, respectively. In addition, use of *Cg*AM on pea starch also gave a similar pattern of LR-CD size ranges without an irregular pattern or peak gap as observed when using raw starch [36]. This observation may be contributed by amylose and amylopectin contents, as well as the structure of substrates. Moreover, incubation of amylose with *Ta*AM produced smallest LR-CDs of CD22 with a major product of CD24–CD27 [26]. In contrast, D-enzyme on amylose produced CD16 as smallest LR-CDs with a major product of CD18–CD22 [41].

In order to enhance the LR-CD production, several strategies have been employed. Addition of organic solvent as much as 15–20% ethanol was reported to increase the amounts of LR-CDs produced by CGTase from *Bacillus* sp.BT3-2 and *Bacillus macerans* up to 30% and 1000%, respectively [60]. Meanwhile, the addition of 10% ethanol and 5–15% DMSO into the reaction of *Cg*AM led to selectively increase in the production of CD33–CD43 by 10–25%, although the overall LR-CD yield was dropped by 20% and 40%, respectively by the addition of ethanol and DMSO [66]. This may be related to the competition or equilibrium between transglycosylation and hydrolysis reactions. It is also worth noting that the overall synthesis of LR-CDs by *Cg*AM was massively reduced in the presence of other alcohols such as 10% methanol, 5% propanol, and 2–5% butanol, while no LR-CD products were detected when 2–5% decanol or acetonitrile was added into the reaction.

Besides that, mutations on AM gene were another way to improve LR-CD production and change the LR-CD size selectivity. The mutations normally would change the enzyme thermostability and catalytic efficiency. For instance, changing a single cysteine in *Sa*AM to serine (C446S) improved the thermostability and resulted in an increase of larger LR-CDs (CD35–CD42) at a long incubation time [31]. On the other hand, improving thermostability of *Cg*AM in the A406V mutant gave higher product yield, especially at higher temperatures and longer incubation times [32]. The mutation had upwardly shifted the optimal temperature and pH, and resulted in higher transglycosylation activity. Meanwhile, changing the tyrosine residue at the loop tip, Y418 in *Cg*AM to Y418A/D/S mutants shifted the principal product from DP range CD29–CD33 to CD36–CD40 [68]. The mutations influenced the substrate entering and subsequently controlled the amount and size of LR-CDs.

Lastly, substrate pretreatment using starch debranching enzymes could be used to increase the amylose content before cyclization reaction, in order to improve the LR-CD production. A recent example was shown by Suksiri and co-workers where starch pretreatment using novel glycogen debranching enzyme from *C. glutamicum* (*Cg*GDE) reduced the LR-CD production time by *Tf*AM to only 3 h and increase the LR-CD conversion yield up to 2-fold [69]. The same approach was demonstrated on pretreatment of different substrates such as amylomaize [70], pea starch [66], sweet potato starch [71] and high amylose corn starch [72] using commercial isoamylase, and an increase in LR-CD yield was obtained.

## 6. Applications of Large-Ring Cyclodextrins

Cyclic oligosaccharides have an advantage over linear oligosaccharides in that they feature hydrophilic exteriors and hydrophobic interiors, which make them capable of accommodating hydrophobic molecules in the cavity. LR-CDs are highly soluble in water and have relatively larger hydrophobic cavity compared to the small ring cyclodextrins (SR-CDs). The aqueous solubility of LR-CDs is more than 100 g/100 mL, which is significantly higher than SR-CDs; for example, the most used β-CD has aqueous solubility of 1.85 g/100 mL [73]. The hydrophobic cavities provide space to form inclusion complexation with guest molecules and offer broad range of applications in the pharmaceutical industry to improve the physicochemical properties of the drugs and increase the stability, solubility, as well as bioavailability, without molecular modifications [74]. Use of LR-CDs in drug delivery systems may offer an advantage over polymer carriers as they are natural materials and generally have excellent biocompatibility, biodegradability, and low toxicity [75]. Recent example of using LR-CDs to improve drug solubility was demonstrated by Ismail and co-workers, the successful increase in solubility of the poorly water soluble domperidone drug was obtained by complexation with the mixture of LR-CDs produced by incubation of *Tf*AM and tapioca starch [76]. The study reported a 3-fold increase in water solubility when domperidone was in complexation with mixture of LR-CDs and 2.7-fold increase when in complex with the purified CD33. On the other hand, LR-CDs produced by 4αGTases from *T. aquaticus* with DP range from 23–45 was reported to form stable complexes in enthalpy-driven and spontaneous manner with several phenolic compounds, such as chlorogenic acid, 3,4-dihydroxy-1-phenylalanine, ρ-coumaric acid, caffeic acid, pyrogallol, 4-methylcatechol, and catechol [77]. Besides that, an extensive molecular dynamic study showed that α-tocopherol (vitamin E) was spontaneously interacted with CD26 and formed stable inclusion complex, where the α-tocopherol was proposed to be covered by 13–14 or 6–10 subunits of CD26 [78]. Evidently, this was supported by another study which showed that LR-CDs synthesized by *Cg*AM was capable of increasing the aqueous solubility of vitamin E acetate during complexation up to 800-fold [63].

Moreover, the large cavity of LR-CDs offers an ability to accommodate many kinds of detergents. This remarkable feature makes LR-CDs an artificial chaperone that facilitates proper folding and prevents aggregation of heterologous proteins during the protein refolding process. For example, LR-CDs with DP 22 to 45, and over 50 produced by *Ta*AM [26] demonstrated the capability of helping refolding the citrate synthase, carbonic anhydrase B, and lysozyme [79]. The LR-CDs work well as stripping agent by effectively stripping the detergents away from the detergent–protein complex, thus promoting protein folding and preventing aggregation. The use of LR-CDs as an artificial chaperone may become an alternative approach to overcome the protein folding problem in protein overexpression, and the good stability with higher resistance against aging makes LR-CDs a better alternative.

Apart from accommodating drugs and detergents, LR-CDs were reported to be effective in gene delivery applications. As recently demonstrated, cationic nanometer-size nanogels consisting of LR-CDs from Ezaki Glico Co., Ltd. were used as a carrier for native CpG DNA to induce cytokine production [80]. The nanogels containing LR-CDs formed a stable complex with the DNA and showed effective cellular uptake with cytokine secretion. Similar applications of LR-CDs were employed by Fujii and co-workers, where they successfully used the self-assembled nanogel of cholesterol-bearing LR-CDs with spermine group as an intra-tumor carrier to deliver vascular endothelial growth factor (VEGF)-specific short interfering RNA (siVEGF) into tumor cells, and effectively suppressed the neovascularization and growth of renal cell carcinomas in mice [81]. The same source of LR-CDs was also reported to be used as a biomaterial to carry plasmid DNA encoding firefly luciferase [82] and siRNA [83]. Cationic LR-CD carriers in both studies were more effectively internalized by the cells compared to the cationic amylose.

## 7. Other Applications of Amylomaltase

Beside LR-CD production, AM has received huge interest due to capability to produce functional oligosaccharides via the intermolecular transglycosylation reaction. To fulfill a huge demand for more healthy food ingredients, *Cg*AM was used to produce maltooligosylsucroses that have anticariogenic property and could become an alternative for sucrose in food or related products [84]. Besides that, AM with combination of transglucosidase, acted on tapioca starch and pea starch has successfully produced isomaltooligosaccharides (IMOs) with prebiotic properties [85,86]. IMOs were also produced by AM from *Thermotoga maritima* in combination with maltogenic amylase, where the product yield increased to 68% and contained relatively larger IMOs [87]. IMOs are non-digestible prebiotic oligosaccharides that stimulate the growth and activity of bifidobacteria in colon and offer health benefit to the host.

AM also has demonstrated promising application in starch processing such as in the production of thermoreversible gel that can replace gelatin as a gelling and viscosifying agent in food product [88,89]. At present, the thermos table 4αGTase from *T. thermophilus* is being used to convert starch into a commercial thermoreversible gel known as EteniaTM [90]. This thermoreversible gel can be cooled and heated repeatedly, dissolving when heated and gelling when cooled. Most currently, AM from *P. aerophilum* was reported to be capable of producing thermoreversible gel with superior stability than 4αGTase from *T. thermophilus* and could be operated at temperature over 70 °C to convert high-amylose starch [23].

## 8. Conclusions

AM can produce LR-CDs by using amylose or starch as substrate via cyclization reaction. The yield and size distribution of LR-CDs are affected by enzyme sources, substrates, and reaction parameters such as pH, temperature, and incubation time. Since LR-CDs are highly soluble in water and could form an inclusion complex with various hydrophobic guest molecules, they are promising solubility enhancers for drugs and could be employed as a delivery system. Moreover, AM can be used to synthesize functional oligosaccharides and thermoreversible starch gels via the intermolecular transglycosylation reaction.

## Figures and Tables

**Table 1:** Optimum conditions for characterized AMs.

Enzyme Name	Source	Optimum Condition												References
		Cyclization			Disproportionation			Hydrolysis			Coupling			
		T °C	pH	Specific Activity (U/mg)	T °C	pH	Specific Activity (U/mg)	T °C	pH	Specific Activity (U/mg)	T °C	pH	Specific Activity (U/mg)	
Archaea														
AM	*Pyrobaculum aerophilum* IM2	N/A	N/A	N/A	95	6.7	450	N/A	N/A	N/A	N/A	N/A	N/A	[23]
AM	*Pyrobaculum calidifontis* A3MU77	N/A	N/A	N/A	80	6.9	690	N/A	N/A	N/A	N/A	N/A	N/A	[24]
Bacteria														
AM	*Aquifex aeolicus* VF5	N/A	N/A	N/A	90	6.6	44.2	N/A	N/A	N/A	N/A	N/A	N/A	[25]
AM	*Corynebacterium glutamicum* ATCC 13032	30	6.0	0.50	30–45	6.0	21.8–44.3	N/A	N/A	0.02–8.05	N/A	N/A	0.03	[32,33,36]
AM	*Streptococcus agalactiae* YZ1605	30	6.0	0.9	40	6.0	54	N/A	N/A	0.05	N/A	N/A	0.19	[31]
AM	*Thermus aquaticus* ATCC 33923	N/A	N/A	N/A	75	5.5–6.0	2.9	N/A	N/A	N/A	N/A	N/A	N/A	[26]
AM	*Thermus filiformis*	70	5.0	0.64	60	6.5	159	N/A	N/A	1.86 × 10^−2^	N/A	N/A	6.91 × 10^−2^	[27]
4αGTase/AM (TBGT)	*Thermus brockianus*	N/A	N/A	N/A	70	6.0	70734	N/A	N/A	N/A	N/A	N/A	N/A	[28]
4αGTase/AM	*Thermus thermophilus*	N/A	N/A	N/A	72–75	5.5–6.3	400	N/A	N/A	N/A	N/A	N/A	N/A	[29]
4αGTase	*Acidothermus cellulolyticus* 11B	N/A	N/A	N/A	75	7.5	N/A	N/A	N/A	N/A	N/A	N/A	N/A	[37]
4αGTase	*Borrelia burgdorferi*	N/A	N/A	N/A	37	5.5	N/A	N/A	N/A	N/A	N/A	N/A	N/A	[34]
4αGTase	*Escherichia coli* str. K-12	N/A	N/A	N/A	28	6.9	9400	N/A	N/A	N/A	N/A	N/A	N/A	[38]
4αGTase	*Saccharophagus degradans* 2-40	N/A	N/A	N/A	35	8.5	N/A	N/A	N/A	N/A	N/A	N/A	N/A	[39]
4αGTase	*Synechocystis* sp. PCC 6803	N/A	N/A	N/A	45	7.0	5.84	N/A	N/A	N/A	N/A	N/A	N/A	[40]
4αGTase	*Thermus scotoductus*	N/A	N/A	N/A	75	7.5	N/A	N/A	N/A	N/A	N/A	N/A	N/A	[30]
Plant														
D-enzyme (*At*DPE1)	*Arabidopsis thaliana*	N/A	N/A	N/A	37	6.0–8.0	N/A	N/A	N/A	N/A	N/A	N/A	N/A	[41]
D-enzyme (*At*DPE2)	*Arabidopsis thaliana*	N/A	N/A	N/A	42	7.0	N/A	N/A	N/A	N/A	N/A	N/A	N/A	[42]
D-enzyme (*Me*DPE1)	*Manihot esculenta Crantz*	N/A	N/A	N/A	37	6.0–8.0	N/A	N/A	N/A	N/A	N/A	N/A	N/A	[41]
D-enzyme (*St*DPE)	*Solanum tuberosum*	N/A	N/A	N/A	45	6.7	47.5	N/A	N/A	N/A	N/A	N/A	N/A	[13,19]
D-enzyme (*Os*DPE1 and *Os*DPE2)	*Oryza sativa* L.	N/A	N/A	N/A	30–39	6.0–7.0	N/A	N/A	N/A	N/A	N/A	N/A	N/A	[43]

N/A: Not available.

**Table 2:** LR-CD production by AMs and D-enzymes

Enzyme	Origin	Substrate	Degree of Polymerization	References
AM	*Streptococcus agalactiae*	Pea starch	DP22–DP50	[31]
AM	*Thermus aquaticus*	Synthetic amylose	DP22–DP > 60	[26]
AM	*Corynebacterium glutamicum*	Pea starch	DP19–DP50	[63]
AM	*Corynebacterium glutamicum*	Tapioca starch	DP22–DP54	[36]
AM	*Thermus filiformis*	Pea starch	DP22–DP60	[27]
AM	*Streptomyces ST66*	Potato amylose	N/A	[65]
4αGTase	*Synechocystis* sp. PCC 6803	Sucrose	DP24–DP284	[64]
4αGTase	*Synechocystis* sp. PCC 6803	Corn starch	DP12–DP36	[40]
4αGTase	*Deinococcus geothermalis*	Amylose	DP5–DP37	[14]
4αGTase	*Aquifex aeolicus*	Amylose	DP16–DP50	[25]
D-enzyme	*Manihot esculenta* Crantz	Potato amylose	DP16–DP > 60	[41]
D-enzyme	*Arabidopsis thaliana* (*At*DPE1)	Potato amylose	DP16–DP50	[41]
D-enzyme	Potato tuber	Synthetic amylose	DP17–DP > 100	[13]

## Data Availability

Not applicable.

## Supplementary Materials

The following are available online, Figure S1: Sequence alignment of various AMs and disproportionation enzymes, Figure S2: LigPlot of Interaction between *Ta*AM and 17 mer glucan.
