# Supplementary material for: Production of Large-Ring Cyclodextrins by Amylomaltases
Source: Molecules. 2022 Feb 21;27(4):1446. doi: 10.3390/molecules27041446 (PMC8875642; doi:10.3390/molecules27041446)
Supplement: Supplementary file 1 [file molecules-27-01446-s001.zip › molecules-1571580-supplementary.pdf]

```
SaAM            ------------------------------------------------------------   0
TaAM            ------------------------------------------------------------   0
AtDPE1          ------------------------------------------------------------   0
D-enzyme        -----------------------------------------MAIHTCFSLIPSSFS-SPK   18
EcAM            MESKR-LDNAALAAGISPNYINAHGKPQSISAETKRRLL---------------------   38
CgAM            MTARRFLNELADLYGVATSYTDYKGAHIEVSDDTLVKILRALGVNLDTSNLPNDDAIQRQ   60

SaAM            ------------------------------------------------------------   0
TaAM            ------------------------------------------------------------   0
AtDPE1          ---MH---HHHHHGKPIPNPLLGLDSTENLY---FQGIDPFTMEVVSS------------   39
D-enzyme        LP-YP---KNTTFQSPIP----KLS-RPTFM---FDRKGSF-------------------   47
EcAM            -DAMHQRTATKVAVTPVPNVMVYTSGKKM--PMVVEGSGEYSWLLTTE------------   83
CgAM            IALFHDRE----FTRPLPPSVVAVEGDELVFPVHVHDGSPADVHIELEDGTQRDVSQVEN   116

SaAM            --------------------------------------------------------MAKK   4
TaAM            --------------------------------------------------------MELP   4
AtDPE1          ---------NSTCLSSISVGEDFPSEYEQ---------------------WLPVPDPESR   69
D-enzyme        ---------QNGTAAVPAVGEDFPIDYAD---------------------WLPKRDPNDR   77
EcAM            --EGTQYKGHVTGGKAFNLPTKLPEGYHTLTLTQDDQRAHCRVIVAPKRCYEPQALLNKQ   141
CgAM            WTAPREIDGIRWGEASFKIPGDLPLGWHKLHLKSNERSAECGLIITPARLSTADKYLDSP   176

SaAM            RASGVLMHITSLPG--DLGIGTFGREAYAFVDFLVETDQKFWQILPLTTTS----FGDSP   58
TaAM            RAFGLLLHPTSLPG--PYGVGVLGREARDFLRFLKEAGGRYWQVLPLGPTG----YGDSP   58
AtDPE1          RRAGVLLHPTSFRG--PHGIGDLGEEAFRFIDWLHSTGCSVWQVLPLVPPD----EGGSP   123
D-enzyme        RRAGILLHPTSFPG--PYGIGDLGPQAFKFLDWLHLAGCSLWQVLPLVPPGKRGNEDGSP   135
EcAM            KLWGACVQLYTLRSEKNWGIGDFGDLKAMLV-DVAKRGGSFIGLNPIHALYPANPESASP   200
CgAM            -RSGVMAQIYSVRSTLSWGMGDFNDLGNLAS-VVAQDGADFLLINPMHAAEPLPPTEDSP   234
                   *   :  :. .    *:* :.         :   .     : *:           **

SaAM            YQSFSAVAGNTHLIDFDLLTLEGFISKDD--------YQNISFGQDPEVVDYAGLFEKRR   110
TaAM            YQSFSAFAGNPYLIDLRPLAERGYVRLED--------PG-----FPQGRVDYGLLYAWKW   105
AtDPE1          YAGQDANCGNTLLISLDELVKDGLLIKDE--------LPQ---PIDADSVNYQTANKLKS   172
D-enzyme        YSGQDANCGNTLLISLEELVDDGLLKMEE--------LPE---PLPTDRVNYSTISEIKD   184
EcAM            YSPSSRRWLNVIYIDVNAVEDFHLSEEAQAWWQLPTTQQTLQQARDADWVDYSTVTALKM   260
CgAM            YLPTTRRFINPIYIRVEDIPEFNQLEIDLRDDIAEMAAEFRERNLTSDIIERNDVYAAKL   294
                *        *   * .  :                              ::       :

SaAM            PVLEKAVKNFLKEERAT--RMLSDF---LQEEKWVTDFAEFMA-----IKEHFGNKALQE   160
TaAM            PALKEAFRGFKEKASPEEREAFAAFRE--REAWWLEDYALFMA-----LKGAHGGLPWNR   158
AtDPE1          PLITKAAKRLIDGN-GELKSKLLDFRNDPSISCWLEDAAYFAA-----IDNTLNAYSWFE   226
D-enzyme        PLITKAAKRLLSSE-GELKDQLENFRRDPNISSWLEDAAYFAA-----IDNSVNTISWYD   238
EcAM            TALRMAWKGFAQRDDEQ-MAAFRQFVAE--QGDSLFWQAAFDALHAQQVKEDEMRWGWPA   317
CgAM            QVLRAIFE--MPRSSER-EANFVSFVQR--EGQGLID--------------------FAT   329
                  :    .             :  *         :

SaAM            WDDKAIIRREEEALAGYRQKLSEVIKYHEVTQYFFYKQWFELKEYANDKG--IQIIGDMP   218
TaAM            WPLP-LRKREEKALREAKSALAEEVAFHAFTQWLFFRQWGALKAEAEALG--IRIIGDMP   215
AtDPE1          WPEP-LKNRHLSALEAIYESQKEFIDLFIAKQFLFQRQWQKVREYARRQG--VDIMGDMP   283
D-enzyme        WPEP-LKNRHLAALEEVYQSEKDFIDIFIAQQFLFQRQWKKVRDYARSKG--ISIMGDMP   295
EcAM            WPEM-YQNVDSPEVRQFCEEHRDDVDFYLWLQWLAYSQFAACWEISQGYEMPIGLYRDLA   376
CgAM            WCAD-RETAQSESVHGTEPDRDELTMFYMWLQWLCDEQLAAAQKRAVDAGMSIGIMADLA   388
                *        .   :        :    .   *::   *       :      : :  *:

SaAM            IYVSADSVEVWTMPELFKLDRDKQPLAIAGVPADDFSDDGQLWGNPIYNWDYHKESDFDW   278
TaAM            IFVAEDSAEVWAHPEWFHLDEEGRPTVVAGVPPDYFSETGQRWGNPLYRWDVLEREGFSF   275
AtDPE1          IYVGYHSADVWANKKHFLLNKKGFPLLVSGVPPDLFSETGQLWGSPLYDWKAMESDQYSW   343
D-enzyme        IYVGYHSADVWANKKQFLLNRKGFPLIVSGVPPDAFSETGQLWGSPLYDWKAMEKDGFSW   355
EcAM            VGVAEGGAETWCDRELYCLK------ASVGAPPDILGPLGQNWGLPPMDPHIITARAYEP   430
CgAM            VGVHPGGADAQNLSHVLAPD------ASVGAPPDGYNQQGQDWSQPPWHPVRLAEEGYIP   442
                : *   ..:.    .    .         *.* *  .  ** *. *           :
```

```
SaAM          WIYRIQSGVKMYDYLRIDHFKGFSDYWEIRGDYQTANDGSWQPAPGPELFA--TIKEKLG  336
TaAM          WIRRLEKALELFHLVRIDHFRGFEAYWEIPASCPTAVEGRWVKAPGEKLFQ--KIQEVFG  333
AtDPE1        WVNRIRRAQDLYDECRIDHFRGFAGFWAVPSEAKVAMVGRWKVGPGKSLFD--AISKGVG  401
D-enzyme      WVRRIQRATDLFDEFRIDHFRGFAGFWAVPSEEKIAILGRWKVGPGKPLFD--AILQAVG  413
EcAM          FIELLRANMQNCGALRIDHVMSMLRLWWIPYGE-TADQGAYVHYPVDDLLSILALESKRH  489
CgAM          WRNLLRTVLRHSGGIRVDHVLGLFRLFVMPRMQ-SPATGTYIRFDHNALVGILALEAELA  501
              :   :.         *:**. .:   : :         * :       *.    :

SaAM          DLPIIAENLGYIDERAERLLAGTGFPGMKIMEFGFYDTTGNSIDIPHNYTE--NTIAYAG  394
TaAM          EVPVLAEDLGVITPEVEALRDRFGLPGMKVLQFAFDDGMEN-PFLPHNYPAHGRVVVYTG  392
AtDPE1        KIKIIAEDLGVITKDVVELRKSIGAPGMAVLQFAFGGGADN-PHLPHNHEV--NQVVYSG  458
D-enzyme      KINIIAEDLGVITEDVVQLRKSIEAPGMAVLQFAFGSDAEN-PHLPHNHEQ--NQVVYTG  470
EcAM          RCMVIGEDLGTVPVEIVGKLRSSGVYSYKVLYFENDHEKT--FRAPKAYPE--QSMAVAA  545
CgAM          GAVVIGEDLGTFEPWVQDALAQRGIMGTSILWFEHSPSQPG-PRRQEEYRP--LALTTVT  558
                 ::.*:** .              .  :: *             . :      :.

SaAM          THDNEVINGWFENLTVEQKAYAENYMRR--------------------------------  422
TaAM          THDNDTTLGWYRTATPHEKAFMARYLAD------------WGIT---------------F  425
AtDPE1        THDNDTIRGWWDTLDQEEKSKAMKYLSI--------------------------------  486
D-enzyme      THDNDTIRGWWDTLPQEEKSNVLKYLSN--------------------------------  498
EcAM          THDLPTLRGYWECGDLTLGKTLGLYPDEVVLRGLYQDRELAKQGLLDALHKYGCLPKRA-  604
CgAM          THDLPPTAGYLEGEHIALRERLGVLNTDPAAEL-AEDLQ-WQAEILDVAASANALPAREY  616
              ***     *:

SaAM          L--------PNEPITETVLRTLYATVSQTTITCMQDLLDKPADSRMNMPNTVG-GNWQWR  473
TaAM          R--------EEEEVPWALMHLGMKSVARLAVYPVQDVLALGSEARMNYPGRPS-GNWAWR  476
AtDPE1        A--------GEDDISWSVIQAAFSSTAQTAIIPMQDILGLGSSARMNTPATEV-GNWGWR  537
D-enzyme      I--------EEEEISRGLIEGAVSSVARIAIIPMQDVLGLGSDSRMNIPATQF-GNWSWR  549
EcAM          -GHKASLMSMTPTLNRGLQRYIAD--SNSALLGLQPEDWLDMAEPVNIPGTS-YQYKNWR  660
CgAM          VGLERDQRGELAELLEGLHTFVAK--TPSALTCVCLVDMVGEKRAQNQPGTTRDMYPNWC  674
                           :   :        :  ::  :            * *         *

SaAM          MRKEDLTENRK---AFLKEITTIYNRGNKLEHHHHHH----------       507
TaAM          LLPGELSPEH---GARLRAMAEATERL--------------------       500
AtDPE1        IPSSTSFDNLETESDRLRDLLSLYGRL--------------------       564
D-enzyme      IPSSTSFDNLDAEAKKLRDILATYGRL--------------------       576
EcAM          RKLSATLESMFADDGVNKLLKDLDRRRRSAHHHHHH-----------       696
CgAM          IPLCDSE-------GNSVLIESLRENELYHRVAKASKRDLEHHHHHH       714
                                 :     .
```

Figure S1. Sequence alignment of various AMs and disproportionation enzymes. Yellow colours represent the catalytic triad residues. The N-terminal region is shown in green, while the dimerization arm is highlighted in cyan.

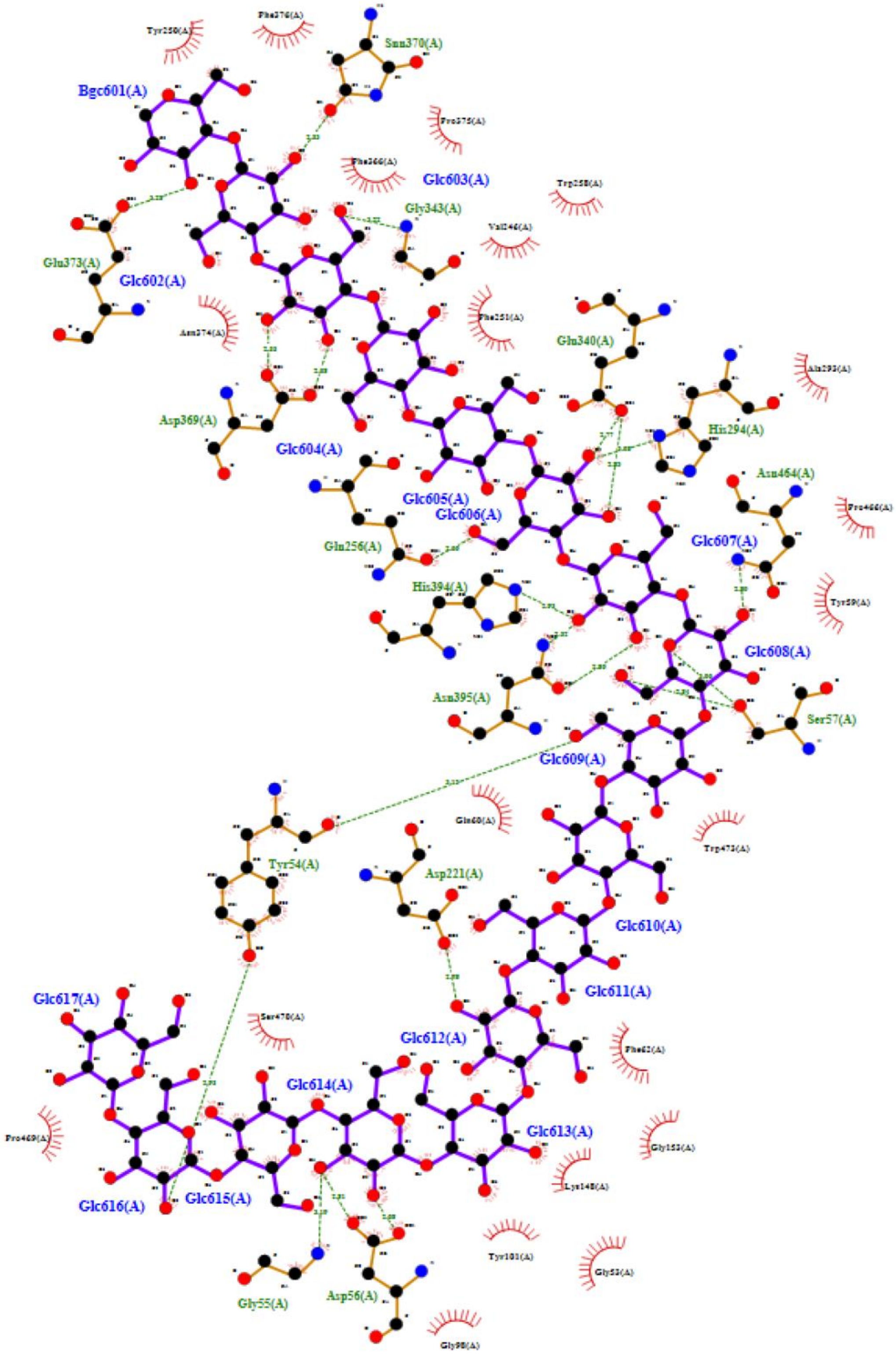

Figure S2. LigPlot of Interaction between *Ta*AM and 17 mer glucan. The structure PDB: 5JIW is analyzed by LigPlot software.
